# Recombinant Rabies Viruses Expressing GM-CSF or Flagellin Are Effective Vaccines for Both Intramuscular and Oral Immunizations

**DOI:** 10.1371/journal.pone.0063384

**Published:** 2013-05-20

**Authors:** Ming Zhou, Guoqing Zhang, Guiping Ren, Clement W. Gnanadurai, Zhenguang Li, Qingqing Chai, Yang Yang, Christina M. Leyson, Wenxue Wu, Min Cui, Zhen F. Fu

**Affiliations:** 1 State Key Laboratory of Agricultural Microbiology, College of Animal Medicine, Huazhong Agricultural University, Wuhan, Hubei, China; 2 Departments of Pathology, University of Georgia, Athens, Georgia, United States of America; 3 Key Laboratory of Zoonosis of Ministry of Agriculture, College of Veterinary Medicine, China Agricultural University, Beijing, China; University of Melbourne, Australia

## Abstract

Our previous studies indicated that recombinant rabies viruses (rRABV) expressing chemokines or cytokines (including GM-CSF) could enhance the immunogenicity by recruiting and/or activating dendritic cells (DC). In this study, bacterial flagellin was cloned into the RABV genome and recombinant virus LBNSE-Flagellin was rescued. To compare the immunogenicity of LBNSE-Flagellin with recombinant virus expressing GMCSF (LBNSE-GMCSF), mice were immunized with each of these rRABVs by intramuscular (i.m.) or oral route. The parent virus (LBNSE) without expression of any foreign molecules was included for comparison. The i.m.-immunized mice were bled at three weeks after the immunization for the measurement of virus neutralizing antibody (VNA) and then challenged with 50 LD_50_ challenge virus standard (CVS-24). Orally immunized mice were boosted after three weeks and then bled and challenged one week after the booster immunization. It was found that both LBNSE-GMCSF and LBNSE-Flagellin recruited/activated more DCs and B cells in the periphery, stimulated higher levels of adaptive immune responses (VNA), and protected more mice against challenge infection than the parent virus LBNSE in both the i.m. and the orally immunized groups. Together, these studies suggest that recombinant RABV expressing GM-CSF or flagellin are more immunogenic than the parent virus in both i.m. and oral immunizations.

## Introduction

Rabies remains a public health threat around the globe and more than 55,000 humans die each year from rabies [1], [2]. Most of the human cases occur in the developing countries of Asia and Africa where canine rabies is endemic [1]. Routine vaccination of dogs is not carried out due to the lack of political will, limited resources and the large population of stray dogs, which are not accessible for parenteral vaccination, resulting in the low coverage of vaccination in dogs [1].In the developed countries, human rabies has been eliminated or reduced to a minimum due to rabies control programs during the past 60 years (routine and mass vaccination of dogs) [1]. However, rabies in wildlife becomes a major threat. It has been reported that more than 90% animal rabies cases occur in wildlife such as raccoons, bats, skunks and foxes in the United States [3], [4]. Bat rabies, particularly the silver-haired bat rabies virus (SHBRV), emerged to be the major source for human infections in the past two decades [5], [6].Therefore, major challenges for rabies control are to immunize stray dogs in the developing countries and wildlife in the developed countries.

Currently inactivated vaccines are used for routine vaccination of pet animals [7], however, multiple immunizations have to be carried out to provide sufficient immunity throughout the life of the animals. Furthermore, vaccination of puppies <3 months of age fails to induce protective immunity, although maternal antibodies declined to undetectable levels by 6 weeks of age [8]. There is a period from the time of the waning maternal antibody to the time of active immunity during which the young animals may not be protected [9]. Most importantly, the inactivated vaccines are expensive to be used in the developing countries and the population of stray dogs is not accessible for any vaccines given parenterally [10]. It is thus important to develop ways for immunizing stray dogs.

Oral rabies vaccines have been successfully developed for wildlife. In the earlier days, an attenuated RABV, Street Alabama Dufferin (SAD) B19, was used in Europe, which resulted in immunization of foxes and stopped RABV spread to untreated areas [11], [12]. However, SAD can cause disease in rodents [13] and domestic animals [14]. Further attenuation of SAD by selecting neutralizing antibody escape mutants resulted in the development of SAG-2 [15], [16] that has been used as vaccine for wildlife in many countries in Europe [16]–[19]. However, a low level of virus-neutralizing antibody (VNA) response has been reported after oral immunization in dogs with SAG-2 [20]. Another widely used oral vaccine for wildlife is the recombinant vaccinia virus expressing RABV G (VRG) [21]. Application of VRG in bait systems resulted in large-scale elimination of fox rabies in parts of Europe [22]. Similar applications of VRG in the United States resulted in a blockade of coyote rabies spread in Texas [23]and raccoon rabies spread in other states [24]–[26]. Although VRG is safe in animals, and efficacious in stimulating active immunity, its exposure to humans can induce intensive skin inflammation and systemic vaccinia infection [27]–[29].Therefore, affordable, safe and efficacious rabies vaccines are needed, particularly for vaccination of stray dogs in the developing countries.

Our previous studies have shown that rRABV expressing chemokines/cytokines including granulocyte-macrophage colony-stimulating factor (GM-CSF), macrophage-derived chemokine (MDC), and macrophage inflammatory protein (MIP-1α),can enhance RABV immunogenicity via recruitment and/or activation of DCs [30]. However, Lee *et al.* demonstrated that despite of the high degree of homology (54%)between the polypeptide of murine GM-CSF and human GM-CSF, the two polypeptides are species specific [31]. In order to overcome possible species specific differences in chemokines and cytokines, bacterial flagellin gene was cloned into RABV to enhance its immunogenicity. Flagellin, the structural component of bacteria flagellar filament, is the ligand for Toll-like Receptor 5 (TLR5) and it can induce the expression of CD80 and CD86 on human immature DCs as well as a variety of chemokines and cytokines such as TNF-alpha, IL-1 beta, and MIP-1 alpha [32]. Because flagellin induces DC maturation [32]–[35], it has been used as a potent systemic and mucosal adjuvant in vaccine development [36]–[43]. In the present study, the gene for bacterial flagellin was cloned in to the genome of the parent virus LBNSE, which was based on SAD B19 strain with two mutations in G protein [30], [44], [45]. This rRABV (LBNSE-Flagellin) was rescued and used to immunize mice in comparison with LBNSE-GMCSF. It was found that both LBNSE-Flagellin and LBNSE-GMCSF recruited/activated more DC and B cells and provided better protection against challenge infection than the parent virus after both intramuscular (i.m.) and oral immunizations.

## Materials and Methods

### Ethics Statement

This study was carried out in strict accordance with the recommendations in the Guide for the Care and Use of Laboratory Animals of the National Institutes of Health. All animal experiments were carried out as approved by the Institutional Animal Care and Use Committee, University of Georgia (animal welfare assurance number: A3085-01). All efforts were made to minimize animal suffering.

### Cells, Viruses, Antibodies, and Animals

BSR cells, a cloned cell line derived from baby hamster kidney (BHK-21) cells, and mouse neuroblastoma (NA) cells [51] were cultured in Dulbecco’s modified Eagle’s medium (DMEM) (Mediatech, Herndon, VA) or RPMI 1640 medium (Mediatech) supplemented with 10% fetal bovine serum (FBS) (Gibco, Grand Island, NY). LBNSE is a recombinant RABV, which is constructed from SAD-B19 strain [44], [45]with mutation of the G at amino acid (aa) positions 194 and 333 [30]. Previous study indicated that with the mutations at amino acid positions 194 and 333 of G protein can result in the virus nonpathogenic for adult mice after intracranial infection [46].LBNSE-GMCSF is a rRABV based on the LBNSE that expresses GM-CSF [30]. Challenge virus standard 11 (CVS-11) was propagated in NA cells, while CVS-24 was propagated in suckling mouse brains. Fluorescein isothiocyanate (FITC)-conjugated antibody against the RABV N protein was purchased from Fujirebio Diagnostics, Inc. (Malvern, PA). Antibodies used in flow cytometric analysis, i.e. anti-CD4 (clone GK1.5), anti-CD8 (clone 53-6.7), anti-CD11b (clone M1/70), anti-CD11c (clone HL3), anti-CD40 (clone 3/23), anti-CD80 (clone 16-10A1), and anti-CD86 (clone GL1),were purchased from BD Pharmingen (San Jose, CA), and anti-CD19(clone 1D3) from eBioscience (San Diego, CA).Female ICR and BALB/c mice were purchased from Harlan and housed in the animal facility of the College of Veterinary Medicine, University of Georgia.

### Construction of LBNSE-Flagellin cDNA Clone

The LBNSE-Flagellin cDNA clone was generated from LBNSE as described previously [30], [47]. Briefly, the vector pLBNSE were digested with BsiWI and NheI between the G and L genes. Flagellin gene was amplified from a plasmid expressing flagellin from Salmonella enterica subsp. Enterica serovar Paratyphi A (kindly supplied by Dr. Margie Lee, Department of Population Health, College of Veterinary Medicine, University of Georgia) and inserted into the vector pLBNSE between these two sites. Two primers were used for amplifying flagellin gene (forward primer: 5′-TGCTCGTACGATGGCACAAGTCATTAAT-3′andreverse primer: 5′-GCTAGCTAGCTTAACGCAGTAAAGAGAG-3′, BsiWI and NheI sites were underlined). The amplified fragment was inserted into the pLBNSE.

### Rescue of rRABV LBNSE-Flagellin

LBNSE-Flagellin was rescued as described previously [30]. Briefly, 2.0 µg of the full-length infectious clone,0.5 µg of N helper plasmid, 0.25 µg of P helper plasmid, 0.1 µg of L helper plasmid and 0.15 µg of G helper plasmid were transfect into BSR cells using the SuperFect transfection reagent (Qiagen, Valencia, CA) according to the manufacturer’s protocol. After 4 days incubation at 34°C, the culture medium was discarded and fresh medium replenished for further incubation (3 more days). Cell culture medium was harvested and tested for rescued virus after reaction with FITC-conjugated anti-RABVN antibody.

### Virus Titration

Virus titration was performed with the direct fluorescent antibody assay (dFA) in NA cells. NA cells in a 96-well plate were inoculated with serial 10-fold dilutions of virus and incubated at 34°C for 48 h. The cell culture medium was discarded, and the cells were fixed with 80%ice-cold acetone for 30 min. The cells were washed twice with PBS and then stained with FITC-conjugated anti-RABV N antibody at 37°C. Antigen-positive foci were counted under a fluorescence microscope (Zeiss, Germany) and virus titer calculated as fluorescent focus units per milliliter (FFU/ml). All titrations were carried out in quadruplicate.

### Mouse Immunization and Challenge Experiment

For intramuscular immunization, groups of 25 ICR mice (Female, 6–8 weeks old) were immunized at the quadriceps muscle with 10^6^ FFU of LBNSE, LBNSE-Flagellin, LBNSE-GMCSF or DMEM in 100 µl volumes. At 21 days after immunization, mice were intracerebrally (i.c.) challenged with 50 mouse intracerebral lethal dose 50 (MICLD_50_) of CVS-24 in 40 µl and observed daily for 3 weeks. For oral immunization, groups of 10 ICR mice (Female, 6–8 weeks old) were given 50 µl of 10^7^ FFU of LBNSE, LBNSE-Flagellin, LBNSE-GMCSF or DMEM into the oral cavity via a needleless syringe. Three weeks post immunization, booster immunization were carried out with the same dose of rRABVs via the oral route. The mice were challenged with 40 µl of 50MICLD_50_ of CVS-24 through i.c. route7 days after booster immunization.

### Western Blotting

NA cells were sham-infected or infected with different viruses and then lysed with RIPA buffer (Thermo Scientific). The lysates were centrifuged at 12,000 rpm for 15 min and the supernatants were mixed with Laemmli Buffer (BIO-RAD).Proteins were resolved by 10% SDS-PAGE, transferred on to a nitrocellulose (NC) membrane, and incubated with mouse anti-Flagellin antibody (Invitrogen, at a dilution of 1∶500)or mouse anti-β-Actin antibody(SIGMA-ALDRICH, at a dilution of 1∶4000). After incubation with goat anti-mouse secondary antibody labeled with horseradish peroxidase (HRP) at dilution of 1∶10000, the NC membrane was incubated with CN/DAB Substrate kit (Thermo Scientific) for color development.

### Rapid Fluorescent Focus Inhibition Test (RFFIT)

Blood was collected from mice for measurement of VNA using the RFFIT as described previously [30]. Briefly, 50 µl of serial five-fold dilutions of serum were prepared in Lab-Tek Chamber slides (NalgeNunc International, Rochester, NY). Fifty FFD50 (50% Fluorescing Foci dose) of CVS-11 was added to each chamber and incubated for 90 min at 37°C. NA cells (10^5^ cells) were added into each chamber and incubated at 37°C for 20 hr. Then the cells were fixed with 80% ice-cold acetone for 30 min and stained with FITC-conjugated anti-RABV N antibody at 37°C for 1 hr. After washing with PBS three times, twenty fields in each chamber were observed under a fluorescent microscope, and the 50% endpoint titers were calculated according to the Reed- Meunch formula [48]. The values were compared with that obtained from the reference serum (purchased from the National Institute for Biological Standards and Control, Herts, UK) and normalized to international units (IU/ml).

### Cultivation of Bone Marrow-derived DCs

Bone marrow-derived DCs were isolated as described previously [30], [49], [50]. Briefly, BALB/c mice were euthanized and bone marrow collected by cutting and collecting the bone between the femur and hip joints. Bone marrow was transferred to a 6-well plate through flushing by a 10-ml syringe loaded with RPMI 1640 and dissociated into single cell suspension. The DC precursors were counted on a hemocytometer and adjusted to a density of 2×10^5^ cells per ml, then cultured in DC medium (RPMI medium containing 0.1% 2-mercaptoethanol, 1×nonessential amino acids, and1×sodium pyruvate) supplemented with 40 ng/ml recombinant mouse GM-CSF.

### Flow Cytometry

Inguinal, cervical, and mesenteric lymph nodes as well as blood were collected from mice (red blood cells in the blood were lysed by ACK Lysing Buffer (BioSource International, Camarillo, CA). Single-cell suspensions were prepared at10^6^ cells/ml in Stain Buffer (BD Pharmingen) and then stained with antibodies against CD40, CD19, CD11b, CD11c, CD80 and CD86 at 4°C for 30 min. After staining with antibodies, cells were washed three times and then fixed with 1% paraformaldehyde. Flow cytometery was performed on LSR-II flow cytometer (BD Bioscience) and data analyzed by BD FACSDiva (BD Pharmingen) and FlowJo software (Tree Star).

### Statistical Analyses

Statistical significance among experimental groups was determined using one-way ANOVA or Fisher’s exact test (χ^2^).

## Results

### Generation and Characterization of rRABV Expressing Flagellin *in vitro*

**Figure 1:**
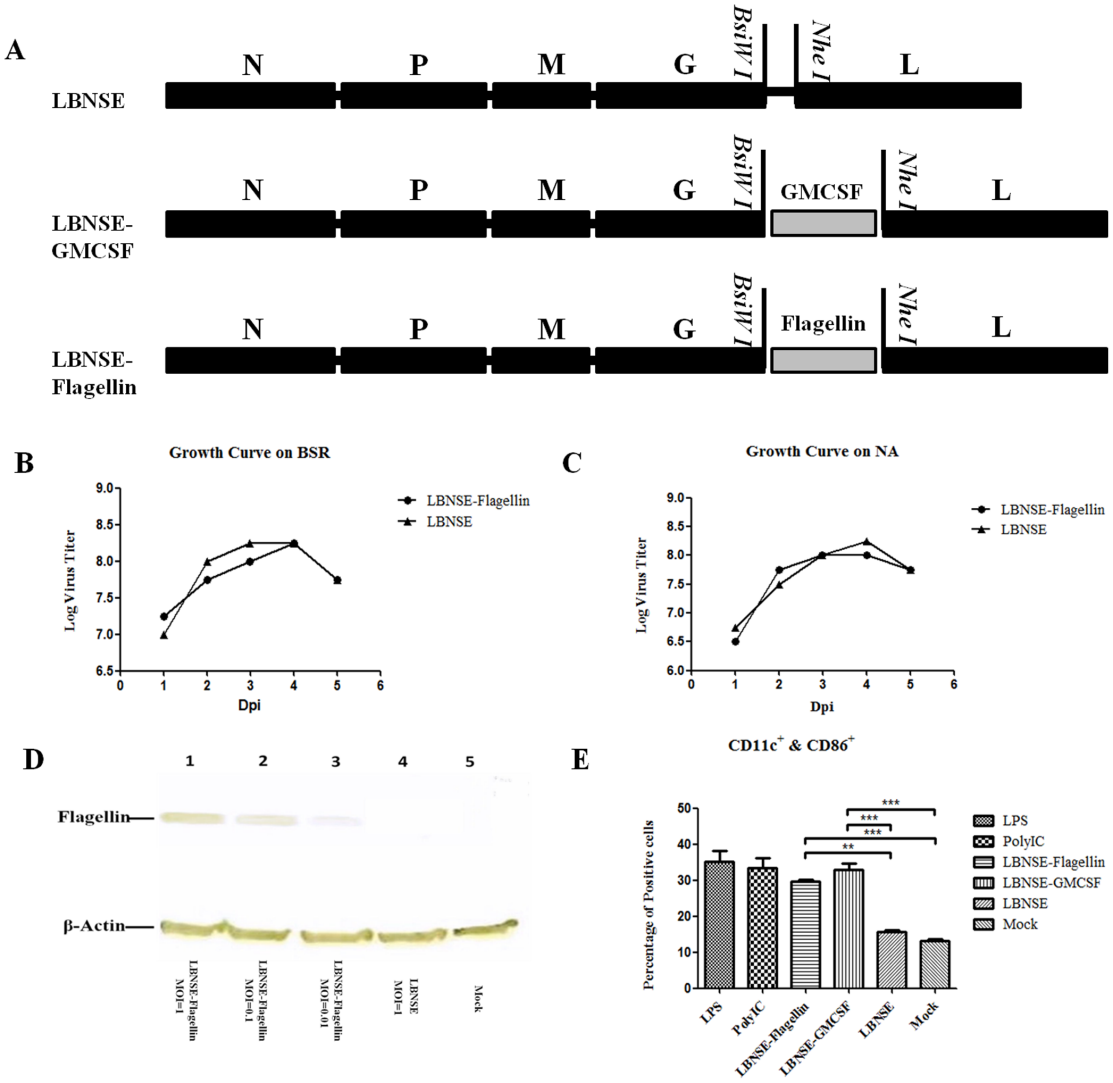
Construction and in vitro characterization of rRABV expressing flagellin. Schematic diagram for the construction of rRABVs LBNSE, LBNSE-GMCSF, and LBNSE–Flagellin (A). The pLBNSE vector was constructed from SAD B19 by deleting the long non-coding region of the G gene and adding BsiWI and NheI sites between the G and L genes [30]. N, P, M,G and L represented RABV nucleoprotein, phosphoprotein, matrix protein, glycoprotein, and polymerase genes, respectively. Mouse GM-CSF [30] and bacterial flagellin genes were individually cloned between the G and L instead of thelong non-coding region of the G gene. Virus growth curves were determined on BSR cells (B) or NA cells (C). Cells were infected with either the LBNSE or LBNSE-Flagellin at a multiplicity of infection (MOI) of 0.01. The culture supernatants were harvested at 1,2,3, 4 and 5 dpi, and viral titers determined as described in Materials and Methods. All titrations were carried out in quadruplicate. The level of flagellin expression was determined by Western blotting analysis (D). NA cells were sham-infected, infected with LBNSE-Flagellin, (MOI = 1, 0.01, or 0.001), or LBNSE (MOI = 1) for 24 hrs, then cells were collected and lysed for Western blotting. The levels ofβ-Actin were assayed as a loading control. Activation of bone marrow-derived DCs by rRABV was determined (E), Bone marrow was harvested from BALB/c mice, and DC precursors were cultured with GM-CSF. The cells were infected with each of the rRABVs. The expression of DC activation markers (CD11c^+^and CD86^+^) was analyzed with flow cytometery. Both LPS and PolyI:C were used as positive controls, and the medium from untreated cells (Mock) was used as negative controls. Data are the means from three independent experiments. The horizontal lines represent the geometric mean for each group, and statistical analysis was performed by one-way ANOVA. (*,P<0.05;**,P<0.01;***,P<0.001).

Our previous studies indicated that over-expression of chemokine MIP-1 alpha [51]and cytokine GM-CSF [30]enhanced the innate and adaptive immune responses by activating and recruiting DC. To overcome the possible species specificity of GM-CSF, the flagellin gene from Salmonella enterica subsp. Enterica serovar Paratyphi was cloned into the LBNSE genome (Fig. 1A) as described previously [30]. The insertion of the flagellin gene was confirmed by sequencing the infectious clone and the rRABV was rescued in BSR cells using the procedures described [52]. The rRABV was designated as LBNSE-Flagellin. In order to characterize LBNSE-Flagellin *in vitro,* viral growth kinetics were examined in BSR and NA cells. As shown in Fig. 1B and 1C, LBNSE-Flagellin grew in both BSR and NA cells as efficiently as the parent strain LBNSE, indicating that viral replication is not affected by the insertion of flagellin gene. The expression of flagellin was detected by Western blotting. Since the lack of a eukaryotic signal sequence in the flagellin gene, flagellin was predicted to be restricted primarily inside the infected cells. As shown in Fig. 1D, no flagellin was expressed in cells sham-infected or infected with LBNSE, while LBNSE-Flagellin expressed flagellin in a dose-dependent manner. To investigate if LBNSE-Flagellin can activate DCs *in vitro* as well as LBNSE-GMCSF [30], bone marrow-derived DCs were isolated from BALB/c mice and co-cultured with each of the rRABVs. LPS and PolyI:C were used as positive controls. As shown in Fig. 1E, both LBNSE-Flagellin and LBNSE-GMCSF activated significantly more DCs than the parent virus LBNSE. The results suggest that LBNSE-Flagellin, similar to LBNSE-GMCSF, can activate bone marrow derived-DCs *in vitro*.

### Recruitment and Activation of DCs and B cells *in vivo* by rRABVs

**Figure 2:**
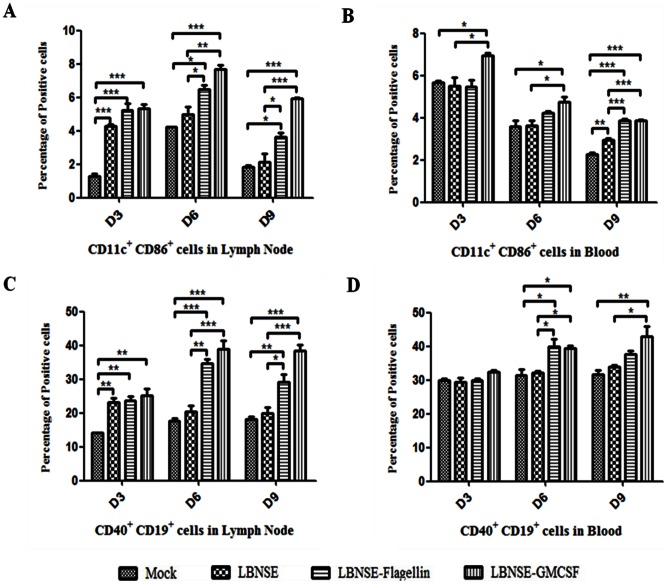
Recruitment and/or activation of DCs and B cells in draining lymph nodes and blood after i.m. inoculation with rRABVs. BALB/c mice were infected with 1×10^6^ FFU of rRABVs or DMEM by i.m. route. The draining (inguinal) lymph nodes and blood were collected on 3, 6 and 9 dpi. Single cell suspensions were prepared from the draining lymph nodes or blood and stained with antibodies for DCs (CD11c^+^ and CD86^+^) (A and B) and B cells (CD19^+^ and CD40^+^) (C and D). Asterisks indicate significant differences between the experimental groups as analyzed by one-way ANOVA. (*,P<0.05;**,P<0.01;***,P<0.001).

Our previous study has shown that rRABV LBNSE-GMCSF can recruit and/or activate DCs and B cells *in vivo* [30]. Flagellin has been reported to stimulate the maturation and activation of human DCs [32], [53]. To investigate if LBNSE-Flagellin can induce the recruitment and maturation of DCs, BALB/c mice were immunized with 10^6^ FFU of rRABV by the i.m. route. Flow cytometric analysis was carried out to detect the recruitment and activation of DCs and B cells in the draining lymph nodes and the blood at 3, 6 and 9 days post infection (dpi).As shown in Fig. 2A and 2C, significantly more DCs (CD86^+^ and CD11c^+^) and B cells (CD40^+^ and CD19^+^) were detected in the draining lymph nodes (inguinal) of mice immunized with LBNSE-Flagellin and LBNSE-GMCSF than in mice immunized with the parent virus LBNSE at 6 and 9 dpi. Significantly more DCs were detected in the blood of mice immunized with LBNSE-GMCSF than in those immunized with the parent virus at all time points (3, 6, 9 dpi), while significantly more DCs were observed in the blood of mice immunized with LBNSE-Flagellin than in mice immunized with the parental virus at 9 dpi(Fig. 2B). Thus the difference in peripheral immune activation indicates that the LBNSE-GMCSF induces early recruitment and/or activation of DCs, whereas, LBNSE-Flagellin shows delayed activation of DCs in the peripheral blood. Significantly more activated B cells were detected in the blood of mice immunized with LBNSE-Flagellin or LBNSE-GMCSF than in mice immunized with the parent virus at 6 dpi, while the significant difference was only found in mice immunized with LBNSE-GMCSF at 9dpi(Fig. 2D). This result indicates LBNSE-GMCSF induces persistent B cell activation, while LBNSE-Flagellin activates B cell transiently. All these data suggest that both LBNSE-GMCSF and LBNSE-Flagellin can recruit and/or activate more DCs and B cells in lymph nodes and peripheral blood than the parent virus.

### VNA Induction and Protection by rRABV via i.m. Immunization

**Figure 3:**
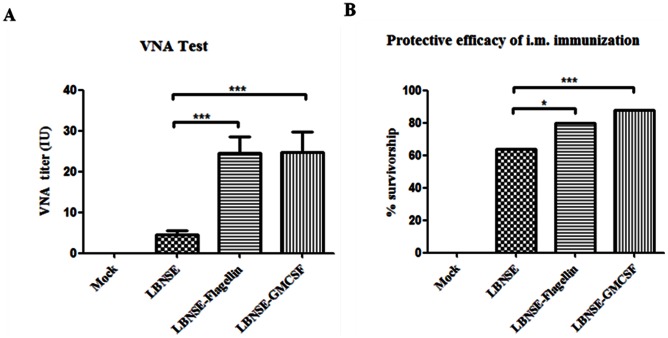
VNA production and protection by rRABVs after i.m. immunization. Groups of 10 ICR mice were immunized with 1×10^6^ FFU of LBNSE, LBNSE-GMCSF, LBNSE-Flagellin, or DMEM by the i.m. route. At 21 days post immunization, blood was collected and serum was separated for VNA test by RFFIT (A).For protection studies, mice (25 in each group) were immunized as above. At 21 days after immunization, immunized mice were challenged with 50 MICLD_50_ of CVS-24 and observed daily for 2 weeks. The numbers of survivors were recorded and compared (B). Asterisks indicate significant differences between the experimental groups as analyzed by one-way ANOVA or Fisher’s exact test (χ^2^).(*,P<0.05;**,P<0.01;***,P<0.001).

In order to determine if the rRABVs induce more VNA production than the parent RABV, mice (10 in each group) were immunized once by the i.m route (hind leg) with 10^6^ FFU of rRABVs. Blood samples were collected at 21 dpi, and serum VNA was determined by RFFIT. Significantly higher VNA titers were detected in mice immunized with LBNSE-GMCSF (24.83±15.79 IU) or LBNSE-Flagellin (24.63±12.32 IU) than in mice immunized with the parent virus (4.60±3.10 IU) (Fig. 3A). To investigate if the higher VNA titer correlates with better protection, mice (n = 25 in each group) immunized with 10^6^ FFU of rRABV by i.m. route and then challenged by i.c. route with 50 MICLD_50_of CVS-24 on day 21 after vaccination. Challenged mice were observed for the development of rabies for 2 weeks. As shown in the Fig. 3B, more survivors were observed in mice immunized with LBNSE-GMCSF or LBNSE-Flagellin than those immunized with the parent virus LBNSE. Overall these results indicate that rRABV expressing GMCSF or flagellin stimulates higher VNA responses and provides better protection than the parent virus.

### Recruitment and/Activation of DC and B cells after Oral Immunization

**Figure 4:**
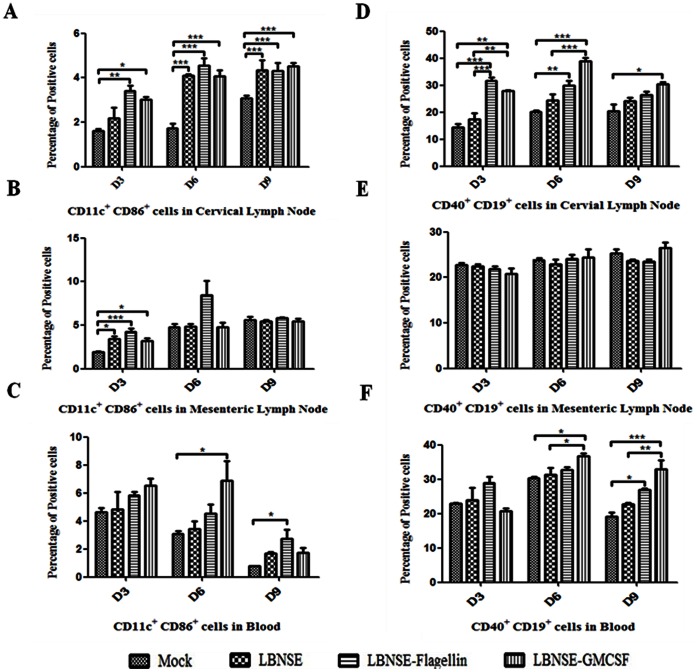
Recruitment and/or activation of DCs and B cells in cervical and mesenteric lymph nodes as well as in blood of mice after primary oral immunization with rRABV. BALB/c mice were orally immunized with 1×10^7^ FFU of rRABVs or DMEM. The cervical and mesenteric lymph nodes as well as the blood were collected on 3, 6 and 9 dpi. Single cell suspensions were prepared and stained with antibodies for DCs (CD11c^+^ and CD86^+^) (A, B and C) and B cells (CD19^+^ and CD40^+^) (D, E and F). Asterisks indicate significant differences between the experimental groups as analyzed by one-way ANOVA. (*,P<0.05;**,P<0.01;***,P<0.001).

To determine if oral immunization with rRABV also recruits and activates DC and B cells, mice were orally immunized with 10^7^ FFU of LBNSE, LBNSE-Flagellin or LBNSE-GMCSF. Sham-immunized (medium only) mice were included as controls. Draining lymph nodes (cervical and mesenteric lymph nodes) and blood were collected on 3, 6, and 9 dpi. Single cell suspensions were prepared and stained with antibodies for DCs and B cells. In the cervical lymph nodes, significantly more DCs were found in mice immunized with LBNSE-Flagellin or LBNSE-GMCSF than sham-immunized mice at 3 dpi. By 6 and 9 dpi, significantly more activated DCs were detected in all the immunized mice than in sham-immunized mice (Fig. 4A). In the mesenteric lymph nodes, significantly more DCs were detected in immunized mice than in sham-immunized mice at 3 dpi, indicating transient early activation of DCs (Fig. 4B). In the blood, significantly more DCs were detected in mice immunized with LBNSE-GMCSF and LBNSE-Flagellin than in sham-immunized mice at 6 dpi and 9 dpi, respectively (Fig. 4C).LBNSE-GMCSF recruited and/or activated significantly more B cells in the cervical lymph nodes than the parent virus at 3 and 6 dpi (Fig. 4D).Significantly more activated B cells were detected at 3 dpi in mice immunized with LBNSE-Flagellin than the parent virus, indicating transient early activation of B cells. No significant difference in B cells activation and recruitment were found in the mesenteric lymph nodes at any time points between these experimental groups (Fig. 4E). In the blood, significantly more B cells were detected in mice immunized with LBNSE-GMCSF than in mice immunized with the parent virus at 6 and 9 dpi (Fig. 4F). Significantly more B cells were detected in mice immunized with LBNSE-Flagellin than in sham-immunized mice at 9 dpi (Fig. 4F). Overall, our data suggest that LBNSE-GMCSF and LBNSE-Flagellin can recruit and activate B cells in the draining lymph nodes, particularly the cervical lymph nodes.

**Figure 5:**
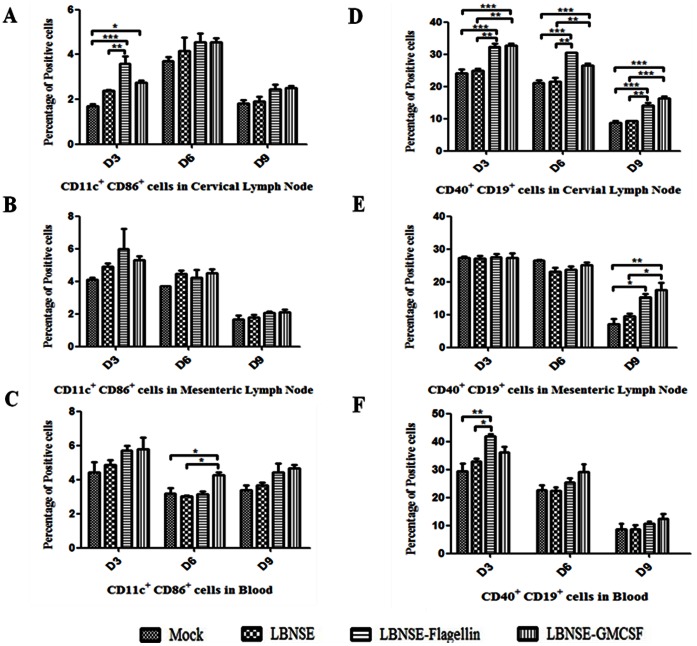
Recruitment and/or activation of DCs and B cells in cervical and mesenteric lymph nodes as well as blood after booster oral immunization. BALB/c mice were orally immunized with 1×10^7^ FFU of rRABVs or DMEM and booster oral immunization was carried out at day 21 after the primary immunization by immunizing with the same dose. Then the cervical and mesenteric lymph nodes as well as the blood were collected on 3, 6 and 9 days after boost immunization. Single cell suspensions were prepared and stained with antibodies for DCs (CD11c^+^ and CD86^+^) (A, B and C) and B cells (CD19^+^ and CD40^+^) (D, E and F). Asterisks indicate significant differences between the experimental groups as analyzed by one-way ANOVA (*,P<0.05;**,P<0.01;***,P<0.001).

After booster oral immunization, significantly more DCs were detected in the cervical lymph nodes in mice immunized with LBNSE-Flagellin or LBNSE-GMCSF than in sham-immunized mice or in mice immunized with the parent virus LBNSE at day 3 after booster immunization, indicating early transient activation of DCs in cervical lymph nodes (Fig. 5A). No significant differences for recruitment and/or activation of DCs were detected in the mesenteric lymph nodes (Fig. 5B). In the blood, LBNSE-GMCSF recruited and/or activated significantly more DCs than the parent virus at day 6 after booster immunization (Fig. 5C).In the cervical lymph nodes, significantly more B cells were detected in mice immunized with LBNSE-GMCSF and LBNSE-Flagellin than in mice immunized with the parent virus at days 3, 6, and 9 after booster immunization (Fig. 5D).In the mesenteric lymph nodes, significantly more B cells were detected in mice immunized with LBNSE-GMCSF and LBNSE-Flagellin than in sham-immunized mice or mice immunized with the parent virus at 9 dpi(Fig. 5E). In the blood, significantly more B cells were detected in mice immunized with LBNSE-Flagellin than in mice immunized with the parent virus (Fig. 5F).All these data indicate that LBNSE-Flagellin and LBNSE-GMCSF can recruit and/or activate more DCs and B cells than parent virus LBNSE after booster immunization.

### VNA Induction and Protection by rRABV via Oral Immunization

**Figure 6:**
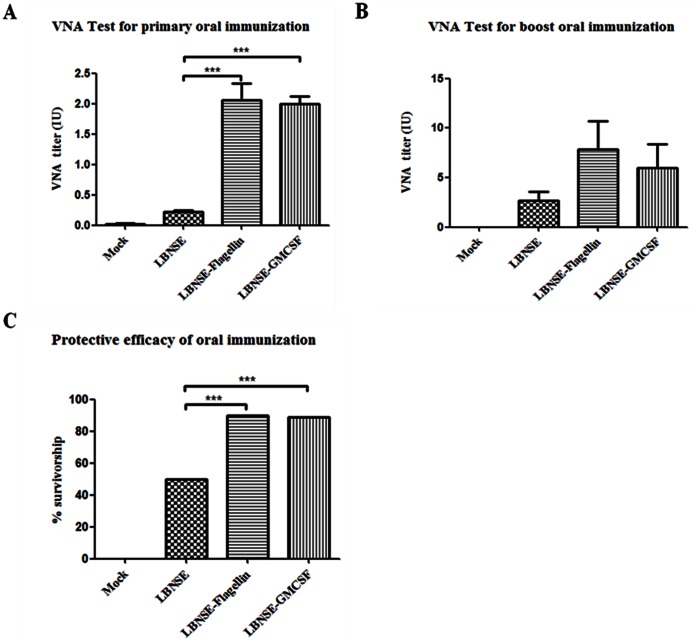
VNA production and protection by rRABVs after oral immunization. Groups of 10 ICR mice were orally immunized with 1×10^7^ FFU of LBNSE, LBNSE-GMCSF, LBNSE-Flagellin or DMEM. At 21 days post primary immunization, blood was collected from mice and serum was separated for VNA test by RFFIT (A). After blood collection, booster oral immunization was carried out with the same dose of rRABVs or DMEM. At day 7 post booster immunization, blood was collected for VNA test (B). Then mice were challenged with 50 MICLD_50_ of CVS-24 and observed daily for 2 weeks, and the numbers of survivors were recorded and compared(C). Asterisks indicate significant differences between the experimental groups as analyzed by one-way ANOVA or Fisher’s exact test (χ^2^). (*,P<0.05;**,P<0.01;***,P<0.001).

To investigate if oral immunization with rRABV induces the production of VNA and provides protection, groups of mice were immunized *per os* with 10^7^ FFU of LBNSE, LBNSE-GMCSF or LBNSE-Flagellin. Sham-immunized mice (medium only) were included as controls. Blood was collected and the serum was used for measurement of VNA at 21 days after primary immunization and the booster immunization (*per os* as well) was carried out at the same time. Blood was collected again at 7 days after booster immunization for VNA test and the mice were challenged with 50 MICLD_50_ of CVS-24 and observed daily for 2 weeks. As shown in Fig. 6A, both LBNSE-GMCSF (2.00±0.27 IU) and LBNSE-Flagellin (2.06±0.60 IU) induced significantly more VNA than the parent virus (0.22±0.04 IU) after primary immunization. After booster immunization, VNA titers were much higher in mice immunized with LBNSE-GMCSF (6.00±5.77 IU) or LBNSE-Flagellin (7.81±7.04 IU) than in mice immunized with the parent virus (2.66±2.30IU) (Fig. 6B). No VNA was detected in the sham-immunized mice. Consistent with the VNA titers in each group, immunization with LBNSE-GMCSF or LBNSE-Flagellin protected almost 90% of the mice while immunization with the parent virus LBNSE provided only 50% protection against a lethal challenge with CVS-24 (Fig. 6C). Overall, our data indicate that both LBNSE-Flagellin and LBNSE-GMCSF can stimulate higher VNA responses and provide better protection than the parent virus after oral vaccination.

### Pathogenicity of rRABVs in Mice

**Figure 7:**
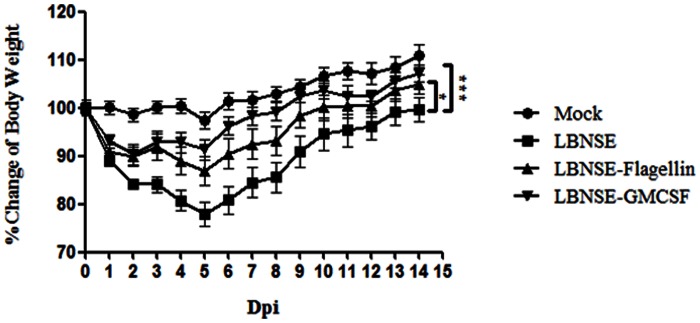
Pathogenicity of rRABVs in mice. Groups of 10 ICR mice (6 to 8 weeks old, female) were infected i.c. with DMEM(mock infection), 1×10^7^ FFU of LBNSE, LBNSE-GMCSF, or LBNSE-Flagellin and their body weights were monitored daily for 2 weeks. Data was obtained from all 10 mice in each group and are presented as mean values± SEM. Asterisks indicate significant differences between the experimental groups as analyzed by one-way ANOVA. (*,P<0.05;**,P<0.01;***,P<0.001).

To determine if expression of GMCSF or Flagellin has adverse effects in mice, four groups of 10 ICR mice(6 to 8 weeks old, female ) were injected with 1×10^7^ FFU of LBNSE, LBNSE-GMCSF, LBNSE-Flagellin or DMEM (mock infection) by the i.c. route and their body weights were measured daily for 2 weeks. As shown in Fig. 7, mice injected with LBNSE-GMCSF and LBNSE-Flagellin lost significantly less body weight than those injected with the parent virus. One of the mice infected with LBNSE lost about 20% body weight and died at 5 dpi. Most of the mice regained their pre-infection body weight by 15 dpi. These data suggest that expression of GMCSF and Flagellin decreased significantly the virulence of RABV.

## Discussion

Our previous studies indicate that expression of cytokines/chemokines including MIP-1α, MDC and GM-CSF can enhance the protective immune responses to RABV via recruitment and/or activation of DCs [30], [50]. Immunization of mice with these recombinant viruses enhanced both innate and adaptive immunity, as demonstrated by higher levels of DC activation and VNA production. The aim of this study is to further confirm the role of DCs in effective immune responses against RABV and to construct additional rRABV that could potentially overcome the species specificity of cytokines/chemokines. Bacterial flagellin, a highly potent adjuvant with the ability to activate DC [36]–[43], [54], was cloned into RABV genome. The immunogenicity of the rRABV expressing flagellin (LBNSE-Flagellin) was determined in a mouse model via i.m. or oral route of immunization in comparison with LBNSE-GMCSF and LBNSE. Our results show that LBNSE-Flagellin, like LBNSE-GMCSF, induced greater activation and maturation of DCs and B cells *in vitro* and *in vivo* than the parent virus [30]. Furthermore, both LBNSE-Flagellin and LBNSE-GMCSF induced higher VNA titers and provided better protection than the parent virus. Thus, LBNSE-flagellin is as effective as LBNSE-GMCSF in activating DC and B cells and protecting mice against challenge infection, yet with the added advantage of overcoming the potential species specificity of cytokines/chemokines. Flagellin is a major structural protein of bacterial flagella and is considered as a pathogen-associated molecular pattern (PAMP). Flagellin binds to TLR5 and subsequently, activates downstream signaling pathways that ultimately results in the production of inflammatory molecules and activation of cellular immune responses, including that of DCs [40]. Flagellin has been cloned into many viral vectors including vesicular stomatitis virus [55], West Nile virus [56], poxvirus [57], and avian influenza virus [58]. In these studies, the addition of flagellin into the viral genomes was found to enhance the efficacy of viral vaccines. Likewise, our findings show that expression of Flagellin gene in RABV vaccine results in increased VNA production and better protection through recruitment and/or activation of DCs and B cells.

Current RABV vaccines for domestic animals (mostly dogs) are inactivated vaccines and given via i.m. route. In the developing countries, dogs are still the primary reservoir for rabies and a large portion of the dog population are stray dogs, not accessible for i.m. vaccination [59]. Thus, oral immunization of stray (free-ranging) dogs would be a very important alternative in controlling rabies in the developing countries [59], [60]. Although oral rabies vaccines (VRG and SAG-2) have been developed successfully and used widely in wildlife animals to eliminate rabies in parts of Europe and to stop rabies spread in North America [61], [62], their use in stray dogs has been limited due to many factors. VRG has been associated with intensive skin inflammation and systemic vaccinia infections in humans [28], raising safety issues since humans are in close contact with dogs. Oral immunization of dogs with live attenuated SAG-2 has been reported to produce only low level of VNA titers [20], making post-vaccination monitoring difficult. Attempts have also been made to develop oral vaccines for dogs using other recombinant viral vectors, such as human [63]or canine adenoviruses [64], [65], pseudorabies virus [66], and parapoxvirus [67]. However, these vaccines induce low VNA responses. A rRABV SPBNGAS-GAS has recently been used for oral immunization in dogs [68].Although it was reported to be very effective, the VNA titers were generally low and not all of the dogs produced detectable VNA after oral immunization. One of the major findings in the present study is that rRABV expressing GM-CSF or flagellin were found to be more immunogenic (higher VNA titers) and to provide better protection than the parent LBNSE (a SAG-2 equivalent) in the mouse model not only via i.m. but more importantly via oral immunization. Although the challenge was conducted after booster immunization in the present study, mice immunized with rRABV developed VNA averaged 2 IU after the first immunization, indicating that these animals would have been adequately protected. On the contrary, mice immunized with the parent virus showed an average VNA below 0.5 IU after the first immunization, generally considered unprotective [69]. These data indicate the superiority of the rRABV over the parent virus in oral immunogenicity.

Investigations for oral vaccination in wildlife indicate that immune responses against RABV are developed in the oral cavity but not necessarily in the gut [70], [71].The oral cavity is rich in lymphatic tissues which could serves as depots for mounting immune responses [72]. Comparatively, the oral cavity has mild physiological conditions. In contrast, the stomach and the intestines are highly acidic and would likely degrade virus particles. Indeed, when comparing immune cells from the cervical lymph nodes (which drain the oral cavity) and the mesenteric lymph nodes (which drain the gut), we observed a greater magnitude of activation of DCs and B cells in the cervical than the mesenteric lymph nodes, which suggest that our rRABV expressing GM-CSF or flagellin most likely initiated immune responses in the oral cavity. Local but limited replication of SAG2 [73] and VRG [72] in the buccal cavity has been demonstrated in orally vaccinated animals, leading to the development of systemic immune responses. In the present study, attempts were made to detect virus in the oral cavity (oral swabs) by virus titration and quantitative reverse transcription-polymerase chain reaction (RT-PCR) (data not shown).Neither virus nor viral RNA was detected in the oral swabs. It is possible that only limited virus replication occurred within the oral cavity, but this is enough to stimulate a robust systemic immune response.

A good vaccine candidate, particularly live-attenuated vaccines, should not only be immunogenic but also safe. Recombinant RABVs expressing GM-CSF or flagellin were found to be further attenuated when compared to the parent LBNSE since significantly lower weight loss was observed in mice infected i.c. with rRABV than in mice with the parent virus LBNSE. This finding is consistent with previous studies with rRABV expressing MIP-1α [51]. Thus, expression of chemokines/cytokines with DC-activation capacity not only enhanced the RABV immunogenicity, but also reduced RABV virulence. Together, the enhancement of immunogenicity, the improvement of protection, and the reduction of rRABV pathogenicity potentiate LBNSE-GMCSF and LBNSE-flagellin to be developed as oral rabies vaccines for dogs. Further studies are warranted to test this contention.

## References

[1] Fu ZF (1997) Rabies and rabies research: past, present and future. Vaccine 15 Suppl: S20–24 10.1016/s0264-410x(96)00312-x 9218287

[2] Knobel DL, Cleaveland S, Coleman PG, Fevre EM, Meltzer MI, et al (2005) Re-evaluating the burden of rabies in Africa and Asia. Bull World Health Organ 83: 360–368. 15976877 PMC2626230

[3] Rupprecht CE, Smith JS, Fekadu M, Childs JE (1995) The ascension of wildlife rabies: a cause for public health concern or intervention? Emerg Infect Dis 1: 107–114. 8903179 10.3201/eid0104.950401 PMC2626887

[4] Nigg AJ, Walker PL (2009) Overview, prevention, and treatment of rabies. Pharmacotherapy 29: 1182–1195. 19792992 10.1592/phco.29.10.1182

[5] Morimoto K, Patel M, Corisdeo S, Hooper DC, Fu ZF, et al (1996) Characterization of a unique variant of bat rabies virus responsible for newly emerging human cases in North America. Proc Natl Acad Sci U S A 93: 5653–5658. 8643632 10.1073/pnas.93.11.5653 PMC39303

[6] Centers for Disease C, Prevention (2012) U.s-acquired human rabies with symptom onset and diagnosis abroad, 2012. MMWR Morb Mortal Wkly Rep 61: 777–781. 23034584

[7] Wu X, Smith TG, Rupprecht CE (2011) From brain passage to cell adaptation: the road of human rabies vaccine development. Expert Rev Vaccines 10: 1597–1608. 22043958 10.1586/erv.11.140

[8] Aghomo HO, Oduye OO, Rupprecht CE (1990) The serological response of young dogs to the Flury LEP strain of rabies virus vaccine. Vet Res Commun 14: 415–425. 2247948 10.1007/BF00343220

[9] Clark KA, Wilson PJ (1996) Postexposure rabies prophylaxis and preexposure rabies vaccination failure in domestic animals. J Am Vet Med Assoc 208: 1827–1830. 8675469

[10] Estrada R, Vos A, De Leon R, Mueller T (2001) Field trial with oral vaccination of dogs against rabies in the Philippines. BMC Infect Dis 1: 23. 11737869 10.1186/1471-2334-1-23 PMC60992

[11] Schneider LG, Cox JH, Muller WW, Hohnsbeen KP (1988) Current oral rabies vaccination in Europe: an interim balance. Rev Infect Dis 10 Suppl 4S654–659. 3206076 10.1093/clinids/10.supplement_4.s654

[12] Wandeler AI, Capt S, Kappeler A, Hauser R (1988) Oral immunization of wildlife against rabies: concept and first field experiments. Rev Infect Dis 10 Suppl 4S649–653. 3206075 10.1093/clinids/10.supplement_4.s649

[13] Winkler WG, Shaddock JH, Williams LW (1976) Oral rabies vaccine: evaluation of its infectivity in three species of rodents. Am J Epidemiol 104: 294–298. 961696 10.1093/oxfordjournals.aje.a112302

[14] Esh JB, Cunningham JG, Wiktor TJ (1982) Vaccine-induced rabies in four cats. J Am Vet Med Assoc 180: 1336–1339. 7096177

[15] Lafay F, Benejean J, Tuffereau C, Flamand A, Coulon P (1994) Vaccination against rabies: construction and characterization of SAG2, a double avirulent derivative of SADBern. Vaccine 12: 317–320. 8178553 10.1016/0264-410x(94)90095-7

[16] Flamand A, Coulon P, Lafay F, Tuffereau C (1993) Avirulent mutants of rabies virus and their use as live vaccine. Trends Microbiol 1: 317–320. 8162418 10.1016/0966-842x(93)90010-o

[17] Cliquet F, Robardet E, Must K, Laine M, Peik K, et al (2012) Eliminating rabies in Estonia. PLoS Negl Trop Dis 6: e1535. 22393461 10.1371/journal.pntd.0001535 PMC3289618

[18] Cliquet F, Aubert M (2004) Elimination of terrestrial rabies in Western European countries. Dev Biol (Basel) 119: 185–204. 15747421

[19] Niin E, Barrat J, Kristian M, Demerson JM, Cliquet F (2006) First oral vaccination of wildlife against rabies in Estonia. Dev Biol (Basel) 125: 145–147. 16878472

[20] Hanlon CA, Niezgoda M, Morrill P, Rupprecht CE (2002) Oral efficacy of an attenuated rabies virus vaccine in skunks and raccoons. J Wildl Dis 38: 420–427. 12038142 10.7589/0090-3558-38.2.420

[21] Kieny MP, Lathe R, Drillien R, Spehner D, Skory S, et al (1984) Expression of rabies virus glycoprotein from a recombinant vaccinia virus. Nature 312: 163–166. 6548799 10.1038/312163a0

[22] Brochier B, Kieny MP, Costy F, Coppens P, Bauduin B, et al (1991) Large-scale eradication of rabies using recombinant vaccinia-rabies vaccine. Nature 354: 520–522. 1758494 10.1038/354520a0

[23] Fearneyhough MG, Wilson PJ, Clark KA, Smith DR, Johnston DH, et al (1998) Results of an oral rabies vaccination program for coyotes. J Am Vet Med Assoc 212: 498–502. 9491156

[24] Hanlon CA, Niezgoda M, Hamir AN, Schumacher C, Koprowski H, et al (1998) First North American field release of a vaccinia-rabies glycoprotein recombinant virus. J Wildl Dis 34: 228–239. 9577769 10.7589/0090-3558-34.2.228

[25] Robbins AH, Borden MD, Windmiller BS, Niezgoda M, Marcus LC, et al (1998) Prevention of the spread of rabies to wildlife by oral vaccination of raccoons in Massachusetts. J Am Vet Med Assoc 213: 1407–1412. 9828930

[26] Roscoe DE, Holste WC, Sorhage FE, Campbell C, Niezgoda M, et al (1998) Efficacy of an oral vaccinia-rabies glycoprotein recombinant vaccine in controlling epidemic raccoon rabies in New Jersey. J Wildl Dis 34: 752–763. 9813845 10.7589/0090-3558-34.4.752

[27] Mempel M, Isa G, Klugbauer N, Meyer H, Wildi G, et al (2003) Laboratory acquired infection with recombinant vaccinia virus containing an immunomodulating construct. J Invest Dermatol 120: 356–358. 12603846 10.1046/j.1523-1747.2003.12074.x

[28] Rupprecht CE, Blass L, Smith K, Orciari LA, Niezgoda M, et al (2001) Human infection due to recombinant vaccinia-rabies glycoprotein virus. N Engl J Med 345: 582–586. 11529212 10.1056/NEJMoa010560

[29] Centers for Disease Control and Prevention (2009) Human vaccinia infection after contact with a raccoon rabies vaccine bait - Pennsylvania, 2009. MMWR Morb Mortal Wkly Rep 58: 1204–1207. 19893480

[30] Wen Y, Wang H, Wu H, Yang F, Tripp RA, et al (2011) Rabies virus expressing dendritic cell-activating molecules enhances the innate and adaptive immune response to vaccination. J Virol 85: 1634–1644. 21106736 10.1128/JVI.01552-10 PMC3028913

[31] Lee F, Yokota T, Otsuka T, Gemmell L, Larson N, et al (1985) Isolation of cDNA for a human granulocyte-macrophage colony-stimulating factor by functional expression in mammalian cells. Proc Natl Acad Sci U S A 82: 4360–4364. 3925454 10.1073/pnas.82.13.4360 PMC390413

[32] Means TK, Hayashi F, Smith KD, Aderem A, Luster AD (2003) The Toll-like receptor 5 stimulus bacterial flagellin induces maturation and chemokine production in human dendritic cells. J Immunol 170: 5165–5175. 12734364 10.4049/jimmunol.170.10.5165

[33] Pino O, Martin M, Michalek SM (2005) Cellular mechanisms of the adjuvant activity of the flagellin component FljB of Salmonella enterica serovar typhimurium to potentiate mucosal and systemic responses. Infection and Immunity 73: 6763–6770. 16177354 10.1128/IAI.73.10.6763-6770.2005 PMC1230971

[34] Didierlaurent A, Ferrero I, Otten LA, Dubois B, Reinhardt M, et al (2004) Flagellin promotes myeloid differentiation factor 88-dependent development of Th2-type response. J Immunol 172: 6922–6930. 15153511 10.4049/jimmunol.172.11.6922

[35] Tsujimoto H, Uchida T, Efron PA, Scumpia PO, Verma A, et al (2005) Flagellin enhances NK cell proliferation and activation directly and through dendritic cell-NK cell interactions. J Leukoc Biol 78: 888–897. 16033815 10.1189/jlb.0105051

[36] McEwen J, Levi R, Horwitz RJ, Arnon R (1992) Synthetic recombinant vaccine expressing influenza haemagglutinin epitope in Salmonella flagellin leads to partial protection in mice. Vaccine 10: 405–411. 1376012 10.1016/0264-410x(92)90071-q

[37] Levi R, Arnon R (1996) Synthetic recombinant influenza vaccine induces efficient long-term immunity and cross-strain protection. Vaccine 14: 85–92. 8821654 10.1016/0264-410x(95)00088-i

[38] McSorley SJ, Ehst BD, Yu Y, Gewirtz AT (2002) Bacterial flagellin is an effective adjuvant for CD4+ T cells in vivo. J Immunol 169: 3914–3919. 12244190 10.4049/jimmunol.169.7.3914

[39] Honko AN, Sriranganathan N, Lees CJ, Mizel SB (2006) Flagellin is an effective adjuvant for immunization against lethal respiratory challenge with Yersinia pestis. Infect Immun 74: 1113–1120. 16428759 10.1128/IAI.74.2.1113-1120.2006 PMC1360354

[40] Pino O, Martin M, Michalek SM (2005) Cellular mechanisms of the adjuvant activity of the flagellin component FljB of Salmonella enterica Serovar Typhimurium to potentiate mucosal and systemic responses. Infect Immun 73: 6763–6770. 16177354 10.1128/IAI.73.10.6763-6770.2005 PMC1230971

[41] Bates JT, Honko AN, Graff AH, Kock ND, Mizel SB (2008) Mucosal adjuvant activity of flagellin in aged mice. Mech Ageing Dev 129: 271–281. 18367233 10.1016/j.mad.2008.01.009 PMC2366812

[42] Lee SE, Kim SY, Jeong BC, Kim YR, Bae SJ, et al (2006) A bacterial flagellin, Vibrio vulnificus FlaB, has a strong mucosal adjuvant activity to induce protective immunity. Infect Immun 74: 694–702. 16369026 10.1128/IAI.74.1.694-702.2006 PMC1346682

[43] Mizel SB, Graff AH, Sriranganathan N, Ervin S, Lees CJ, et al (2009) Flagellin-F1-V Fusion Protein Is an Effective Plague Vaccine in Mice and Two Species of Nonhuman Primates. Clinical and Vaccine Immunology 16: 21–28. 18987167 10.1128/CVI.00333-08 PMC2620661

[44] Rasalingam P, Rossiter JP, Mebatsion T, Jackson AC (2005) Comparative pathogenesis of the SAD-L16 strain of rabies virus and a mutant modifying the dynein light chain binding site of the rabies virus phosphoprotein in young mice. Virus Res 111: 55–60. 15896402 10.1016/j.virusres.2005.03.010

[45] Conzelmann KK, Cox JH, Schneider LG, Thiel HJ (1990) Molecular cloning and complete nucleotide sequence of the attenuated rabies virus SAD B19. Virology 175: 485–499. 2139267 10.1016/0042-6822(90)90433-r

[46] Faber M, Faber ML, Li J, Preuss MA, Schnell MJ, et al (2007) Dominance of a nonpathogenic glycoprotein gene over a pathogenic glycoprotein gene in rabies virus. J Virol 81: 7041–7047. 17459937 10.1128/JVI.00357-07 PMC1933278

[47] Schnell MJ, Mebatsion T, Conzelmann KK (1994) Infectious rabies viruses from cloned cDNA. EMBO J 13: 4195–4203. 7925265 10.1002/j.1460-2075.1994.tb06739.x PMC395346

[48] Reed LJ, H Muench (1938) A simple method of estimating fifty percent end points. Am J Hyg 27: 493–497.

[49] Gilboa E (2007) DC-based cancer vaccines. J Clin Invest 117: 1195–1203. 17476349 10.1172/JCI31205 PMC1857263

[50] Lutz MB, Kukutsch N, Ogilvie AL, Rossner S, Koch F, et al (1999) An advanced culture method for generating large quantities of highly pure dendritic cells from mouse bone marrow. J Immunol Methods 223: 77–92. 10037236 10.1016/s0022-1759(98)00204-x

[51] Zhao L, Toriumi H, Wang H, Kuang Y, Guo X, et al (2010) Expression of MIP-1alpha (CCL3) by a recombinant rabies virus enhances its immunogenicity by inducing innate immunity and recruiting dendritic cells and B cells. J Virol 84: 9642–9648. 20592092 10.1128/JVI.00326-10 PMC2937656

[52] Inoue K, Shoji Y, Kurane I, Iijima T, Sakai T, et al (2003) An improved method for recovering rabies virus from cloned cDNA. J Virol Methods 107: 229–236. 12505638 10.1016/s0166-0934(02)00249-5

[53] Agrawal S, Agrawal A, Doughty B, Gerwitz A, Blenis J, et al (2003) Cutting edge: different Toll-like receptor agonists instruct dendritic cells to induce distinct Th responses via differential modulation of extracellular signal-regulated kinase-mitogen-activated protein kinase and c-Fos. J Immunol 171: 4984–4989. 14607893 10.4049/jimmunol.171.10.4984

[54] Mizel SB, Graff AH, Sriranganathan N, Ervin S, Lees CJ, et al (2009) Flagellin-F1-V fusion protein is an effective plague vaccine in mice and two species of nonhuman primates. Clin Vaccine Immunol 16: 21–28. 18987167 10.1128/CVI.00333-08 PMC2620661

[55] Ahmed M, Puckett S, Arimilli S, Braxton CL, Mizel SB, et al (2010) Stimulation of human dendritic cells by wild-type and M protein mutant vesicular stomatitis viruses engineered to express bacterial flagellin. J Virol 84: 12093–12098. 20844045 10.1128/JVI.00406-10 PMC2977888

[56] McDonald WF, Huleatt JW, Foellmer HG, Hewitt D, Tang J, et al (2007) A West Nile virus recombinant protein vaccine that coactivates innate and adaptive immunity. J Infect Dis 195: 1607–1617. 17471430 10.1086/517613

[57] Delaney KN, Phipps JP, Johnson JB, Mizel SB (2010) A recombinant flagellin-poxvirus fusion protein vaccine elicits complement-dependent protection against respiratory challenge with vaccinia virus in mice. Viral Immunol 23: 201–210. 20374000 10.1089/vim.2009.0107 PMC2883514

[58] Liu G, Song L, Reiserova L, Trivedi U, Li H, et al (2012) Flagellin-HA vaccines protect ferrets and mice against H5N1 highly pathogenic avian influenza virus (HPAIV) infections. Vaccine 30: 6833–6838. 23000130 10.1016/j.vaccine.2012.09.013

[59] Meslin FX, Fishbein DB, Matter HC (1994) Rationale and prospects for rabies elimination in developing countries. Curr Top Microbiol Immunol 187: 1–26. 7859487 10.1007/978-3-642-78490-3_1

[60] Charlton KM, Artois M, Prevec L, Campbell JB, Casey GA, et al (1992) Oral rabies vaccination of skunks and foxes with a recombinant human adenovirus vaccine. Arch Virol 123: 169–179. 1550495 10.1007/BF01317147

[61] Stohr K, Meslin FM (1996) Progress and setbacks in the oral immunisation of foxes against rabies in Europe. Vet Rec 139: 32–35. 8839488 10.1136/vr.139.2.32

[62] MacInnes CD, Smith SM, Tinline RR, Ayers NR, Bachmann P, et al (2001) Elimination of rabies from red foxes in eastern Ontario. J Wildl Dis 37: 119–132. 11272485 10.7589/0090-3558-37.1.119

[63] Vos A, Neubert A, Pommerening E, Muller T, Dohner L, et al (2001) Immunogenicity of an E1-deleted recombinant human adenovirus against rabies by different routes of administration. J Gen Virol 82: 2191–2197. 11514729 10.1099/0022-1317-82-9-2191

[64] Li J, Faber M, Papaneri A, Faber ML, McGettigan JP, et al (2006) A single immunization with a recombinant canine adenovirus expressing the rabies virus G protein confers protective immunity against rabies in mice. Virology 356: 147–154. 16938327 10.1016/j.virol.2006.07.037

[65] Yuan ZG, Li XM, Mahmmod YS, Wang XH, Xu HJ, et al (2009) A single immunization with a recombinant canine adenovirus type 2 expressing the seoul virus Gn glycoprotein confers protective immunity against seoul virus in mice. Vaccine 27: 5247–5251. 19583957 10.1016/j.vaccine.2009.06.062

[66] Yuan Z, Zhang S, Liu Y, Zhang F, Fooks AR, et al (2008) A recombinant pseudorabies virus expressing rabies virus glycoprotein: safety and immunogenicity in dogs. Vaccine 26: 1314–1321. 18262313 10.1016/j.vaccine.2007.12.050

[67] Amann R, Rohde J, Wulle U, Conlee D, Raue R, et al.. (2012) A new rabies vaccine based on a recombinant Orf virus (Parapoxvirus) expressing the rabies virus glycoprotein. J Virol. 10.1128/JVI.02470-12 PMC3554190 23175365

[68] Rupprecht CE, Hanlon CA, Blanton J, Manangan J, Morrill P, et al (2005) Oral vaccination of dogs with recombinant rabies virus vaccines. Virus Res 111: 101–105. 15896409 10.1016/j.virusres.2005.03.017

[69] Monaco F, Franchi PM, Lelli R (2006) Studies on an inactivated vaccine against rabies virus in domestic animals. Dev Biol (Basel) 125: 233–239. 16878481

[70] Black JG, Lawson KF (1973) Further studies of sylvatic rabies in the fox (Vulpes vulpes). Vaccination by the oral route. Can Vet J 14: 206–211. 4755271 PMC1696205

[71] Baer GM, Broderson JR, Yager PA (1975) Determination of the site of oral rabies vaccination. Am J Epidemiol 101: 160–164. 1124754 10.1093/oxfordjournals.aje.a112080

[72] Rupprecht CE, Hamir AN, Johnston DH, Koprowski H (1988) Efficacy of a vaccinia-rabies glycoprotein recombinant virus vaccine in raccoons (Procyon lotor). Rev Infect Dis 10 Suppl 4S803–809. 3206091 10.1093/clinids/10.supplement_4.s803

[73] Orciari LA, Niezgoda M, Hanlon CA, Shaddock JH, Sanderlin DW, et al (2001) Rapid clearance of SAG-2 rabies virus from dogs after oral vaccination. Vaccine 19: 4511–4518. 11483278 10.1016/s0264-410x(01)00186-4

